# Truncated Isoforms of lncRNA ANRIL Are Overexpressed in Bladder Cancer, But Do Not Contribute to Repression of INK4 Tumor Suppressors

**DOI:** 10.3390/ncrna1030266

**Published:** 2015-12-17

**Authors:** Michèle J. Hoffmann, Judith Dehn, Johanna Droop, Günter Niegisch, Christian Niedworok, Tibor Szarvas, Wolfgang A. Schulz

**Affiliations:** 1 Department of Urology, Medical Faculty, Heinrich-Heine-University Düsseldorf, Germany; Moorenstrasse 5, Düsseldorf 40225, Germany; E-Mails: judith.heubach@hhu.de (J.D.); johanna.droop@hhu.de (J.D.); guenter.niegisch@med.uni-duesseldorf.de (G.N.); wolfgang.schulz@hhu.de (W.A.S.); 2 Department of Urology, University of Duisburg-Essen, Hufelandstrasse 55, Essen 45147, Germany; E-Mails: Christian.Niedworok@uk-essen.de (C.N.); sztibusz@gmail.com (T.S.); 3 Department of Urology, Semmelweis University, Ülloi ut 78/b, 1082 Budapest, Hungary

**Keywords:** long noncoding RNA, ANRIL, p16, p14, p15, INK4/ARF, tumor suppressor, polycomb, bladder cancer, PVT1

## Abstract

The *INK4*/*ARF* locus at chromosome 9p21 encoding *p14^ARF^*, *p15^INK4B^* and *p16^INK4A^* is a major tumor suppressor locus, constituting an important barrier for tumor growth. It is frequently inactivated in cancers, especially in urothelial carcinoma (UC). In addition to deletions and DNA hypermethylation, further epigenetic mechanisms might underlie its repression. One candidate factor is the long noncoding RNA ANRIL, which recruits Polycomb proteins (PcG) to regulate expression of target genes *in cis* and *trans*. We observed ANRIL overexpression in many UC tissues and cell lines mainly resulting from upregulation of 3’-truncated isoforms. However, aberrant ANRIL expression was neither associated with repression of *INK4*/*ARF* genes nor with proliferation activity or senescence. We wondered whether truncated ANRIL isoforms exhibit altered properties resulting in loss of function *in cis*. We excluded delocalization and performed RNA immunoprecipitation demonstrating interaction between full length or truncated ANRIL and PcG protein CBX7, but not SUZ12 of PRC2. Our data indicate that ANRIL in UC cells may not interact with PRC2, which is central for initializing gene repression. Thus, tissue-specific binding activities between ANRIL and PcG proteins may determine the regulatory function of ANRIL. In conclusion, ANRIL does not play a major role in repression of the *INK4*/*ARF* locus in UC.

## 1. Introduction

The *INK4*/*ARF* locus at chromosome 9p21 encodes three tumor suppressors (TS), the two cyclin-dependent kinase inhibitors *p15^INK4B^* and *p16^INK4A^*, as well as *p14^ARF^*, a regulator of p53 activity. These proteins are involved in regulation of the cell cycle and other key cellular processes, impinging on replicative senescence, apoptosis and self-renewal of stem cells [1,2]. Thus, active expression of this locus constitutes an important barrier for tumor growth. Consequently, it represents one of the most frequently altered regions in a variety of human cancers, including urothelial carcinoma (UC) [3,4,5,6]. Inactivation in cancer can occur through homozygous deletion, point mutation or DNA hypermethylation in promoter regions [7,8].

In stem cells, the locus is instead repressed by epigenetic mechanisms, especially through histone modifications mediated by Polycomb Group proteins (PcG) [9,10,11]. However, the mechanisms underlying recruitment of PcG proteins to their target genes are still under intense investigation [12]. Only recently, a new class of epigenetic regulatory factors, the long noncoding RNAs (lncRNA), has been discovered. These are expressed in a highly tissue-dependent manner and exert a variety of functions in regulation of transcription, chromatin remodeling or post-transcriptional processing by various mechanisms. For individual lncRNAs, interactions with PcG proteins, organized in different Polycomb Repressive Complexes (PRC), have been reported indicating that they may mediate PcG recruitment to target genes in a tissue-dependent manner [13,14]. Interestingly, the *INK4*/*ARF* locus encodes such a regulatory lncRNA named ANRIL, which has been shown to interact with the PcG proteins CBX7 in the prostate cancer cell line PC-3 [15] and SUZ12 in normal lung fibroblasts [16], respectively. Accordingly, ANRIL may be involved in transcriptional regulation of this tumor suppressor locus *in cis*. *Trans* effects of ANRIL at other genes appear to be mediated by several Alu-like sequences distributed within the lncRNA allowing interactions with Alu sequences across the genome [17].

**Figure 1:**
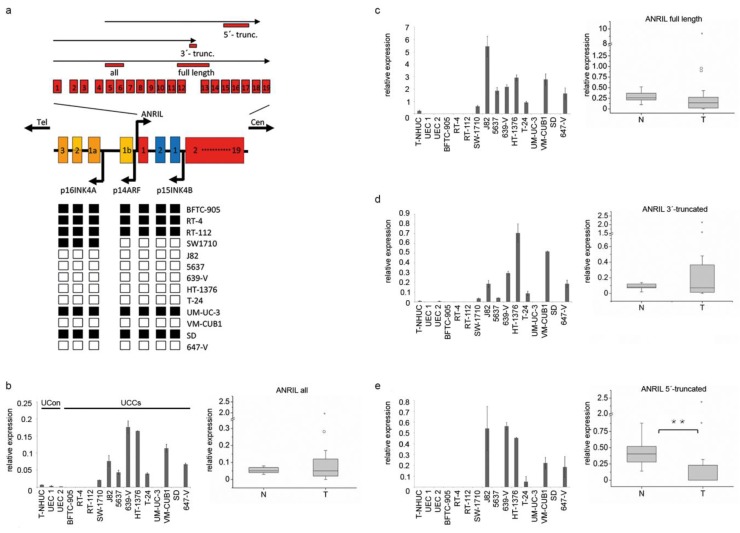
(**a**) Illustration of the *INK4/ARF* locus on chromosome 9p21 encoding for the three tumor suppressors (TS) *p14^ARF^, p15INK4B, p16^INK4A^* and for the lncRNA ANRIL. ANRIL and *p14* share a bidirectional promoter. The full length ANRIL transcript contains 19 exons. Black arrows on top represent different groups of truncated variants and the localization of primer assays is given by red bars. Black and white boxes at the bottom represent the exons of the three TS. Black boxes denote deletions as determined previously by Florl *et al.* [4], white boxes display retentions. (**b**) ANRIL expression across all kind of isoforms was determined in UC cell lines (left graph, bar chart) and tissues (right graph, boxplot representation) with the “all” assay by quantitative RT-PCR. Expression was normalized to the *TBP* reference gene. The cell lines comprised 13 UC cell lines (UCCs) and three normal controls (UCon), one immortalized normal cell line (TERT-NHUC) and two different primary cultures of normal uroepithelial cells (UEC). The tissue sample set 1 comprises 19 UC tissues (T) and 10 normal bladder tissues (N). In an analogous fashion, expression of full length variants (**c**), a group of 3’-truncated (**d**) and a group of 5’- truncated isoforms (**e**) was determined in the cell lines and tissues. (** *p* ≤ 0.01).

ANRIL was first identified by genetic analysis of familial melanoma patients with neural system tumors [18]. Further genome-wide association studies for ANRIL revealed a relationship between genetic polymorphisms, somatic alterations in its structure, or expression changes and various diseases such as coronary artery disease [19], as well as different cancer types, e.g., esophageal, gastric, hepatocellular and lung cancer [20,21,22,23]. As the *INK4*/*ARF* locus is regarded as a major tumor suppressor locus in UC and alterations are highly prevalent, in particular homozygous deletions (Figure 1a), data on expression and function of lncRNA ANRIL are of great interest.

ANRIL is normally transcribed from the bidirectional *p14* promoter in antisense orientation to the protein-coding transcripts from the *INK4*/*ARF* locus. In addition to the initially described 3.8-kb full length transcript [18], a number of alternatively spliced isoforms have been described and shown to be functional [17,19,24], including several that lack terminal exons (Figure 1a). Alternative splicing and thereby the abundance of ANRIL transcript variants appear to be tissue dependent [25]. Intriguingly, even the association of ANRIL with disease risk appears to be isoform-specific [17,24]. In particular, truncated ANRIL isoforms may differ in their abilities to interact with PcGs (or other interacting proteins) and to regulate expression of target genes [17]. As a consequence, the functional role of ANRIL in one cell type cannot be straightforwardly inferred from investigations of other cell types. Accordingly, negative or positive correlations between the expression of ANRIL and that of the three *INK4*/*ARF* TS have been variously observed for a number of tissues or pathological conditions [15,16,18,24,26]. Obviously, further research is required to understand the regulation and function of ANRIL isoforms. One particular open question is which isoforms are expressed in cancer cells carrying deletions in the *INK4*/*ARF* locus and whether these retain any ANRIL functions.

Expression of ANRIL is regulated by E2F transcription factors [23,26,27]. They likely act at the bidirectional promoter shared by ANRIL and *ARF*, which contains E2F binding sites and can be transactivated by E2F1 in reporter assays. From the E2F family, E2F1 and E2F3 are commonly overexpressed in UC, often as a consequence of gene amplifications [28,29,30,31]. In particular, overexpression of E2F3 is found in UC tissues and cell lines with mutations inactivating RB1. Whether E2F3 regulates ANRIL in the same manner as E2F1 is unknown.

To our knowledge, this is the first study investigating the expression of different ANRIL isoforms in any cancer type. We determined expression levels of the three TS *p16^INK4A^*, *p15^INK4B^*, *p14^ARF^* as well as of *E2F1* and *E2F3* in UC cell lines and tissues to assess the mechanisms leading to aberrant expression of ANRIL isoforms. Furthermore, we sought to elucidate the effect of ANRIL isoforms on the adjacent tumor suppressor locus in UC, in particular that of the truncated isoforms.

## 2. Results

### 2.1. 3’-Truncated Isoforms of ANRIL Are Overexpressed in UC

Initially, we determined the expression of different ANRIL isoforms in UC by quantitative real time RT-PCR. To this end, we designed one primer pair detecting all isoforms (Figure 1a “all”, Figure 1b), and other primer pairs specific for major isoform groups with distinct transcription starts and ends including the full length version (Figure 1c), a group of 3’-truncated (Figure 1d) or a group of 5’-truncated variants (Figure 1e), respectively. Thus, our assays for expression analysis covered all kind of ANRIL variants that were annotated in common public databases at that time and have previously been described by Holdt *et al.* [17].

In normal urothelial cells (UCon), all ANRIL isoforms were undetectable (primary UEC cultures) or expressed at extremely low levels (in the immortalized TERT-NHUC line). In UC cell lines with larger homozygous deletions that were mapped earlier by our group (Figure 1a black boxes) [4] no ANRIL expression was detected. All other UC cell lines expressed well detectable levels of ANRIL overall and of the full-length transcript. Cell lines with defective RB1, such as J82, 647-V, 639-V, HT-1376 and 5637 (Table S1) had generally higher expression than cell lines with mutations (VM-CUB1), hypermethylation (T-24) or smaller deletions (SW-1710) of the *INK*/*ARF* locus. Notably, several of these cell lines lacked 5’-truncated transcripts. In some cell lines that do not contain deletions at 9p21, e.g., 639-V and HT-1376, high expression levels across all ANRIL isoforms appeared to result from overexpression of truncated isoforms, but not from the full length transcript.

Similarly, in UC tissues, overall ANRIL expression was upregulated in many cases and individual tumor samples displayed highly increased expression (Figure 1b). Interestingly, expression of the full length transcript was rather reduced in tumors (Figure 1c) as was the expression of the 5’- truncated variants (Figure 1e; *p* ≤ 0.001). Thus, increased levels of overall ANRIL expression in UC tissues resulted from overexpression of 3’-truncated transcripts in about 50% of the patients from sample set 1 (Figure 1d). Similarly, 3’-truncated ANRIL variants were also overexpressed in about 50% of the patients compared to the mean expression of normal control samples in a second large sample cohort comprising 108 UC tissues (Set 2; Figure S1a). However, overexpression of 3’-truncated isoforms in UC was neither significantly correlated with clinicopathologic parameters nor with survival in this larger tissue set (Figure S1b).

### 2.2. ANRIL Expression in UC Can Be Stimulated by E2F1, But Not by E2F3

**Figure 2:**
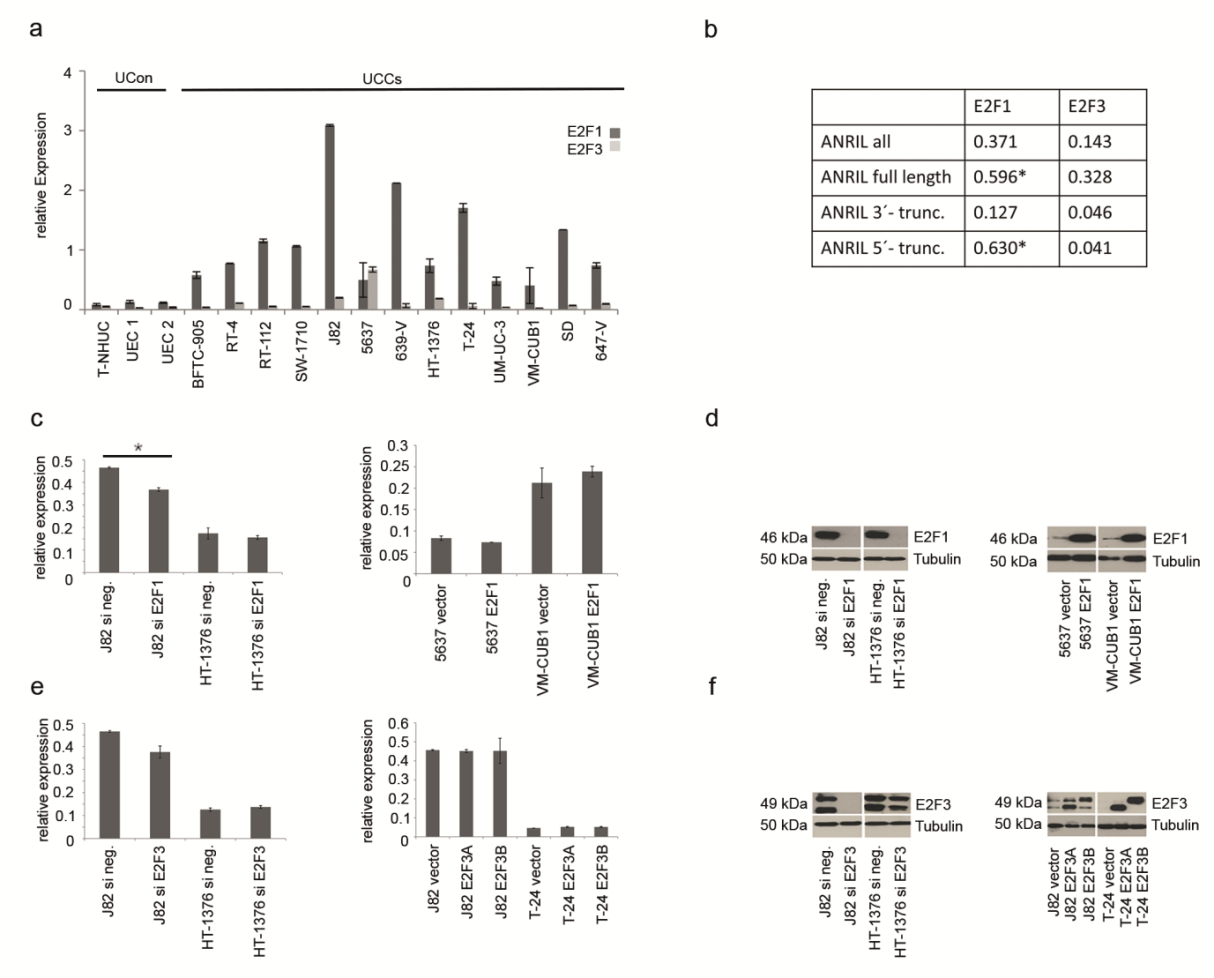
(**a**) mRNA expression of *E2F1* (dark grey bars) and *E2F3* (light grey bars) was determined in UCCs and UCon by qRT-PCR and normalized to *TBP*. (**b**) Expression levels of *E2F* factors in UCCs and controls were correlated with that of ANRIL transcripts by calculating the Pearson correlation coefficient (* *p* ≤ 0.05). (**c**) Relative overall ANRIL expression (“ANRIL all”) was determined by qRT-PCR after siRNA-mediated knockdown of *E2F1* (left panel; * *p* ≤ 0.05) or transient ectopic expression (right panel). Efficiency of *E2F1* knockdown and overexpression was verified on the protein level (**d**). Similarly, ANRIL expression was measured after siRNA-mediated knockdown of *E2F3* (left panel) or transient ectopic expression (right panel). Again, expression modulation of E2F3 was checked on the protein level (**e**). The two different isoforms of *E2F3* (A and B) were overexpressed separately by the respective plasmid. However, the siRNA targeted both isoforms as shown in (**f**). The siRNA knockdown in HT-1376 cells was less efficient.

To investigate the regulation of ANRIL expression by E2F transcription factors in UC, we measured the expression of *E2F1* and *E2F3* in UC cell lines. According to qRT-PCR data, *E2F1* was clearly overexpressed in all UC cell lines compared to normal controls (Figure 2a, dark grey bars). In keeping with previously published results [31,32], *E2F3* was mainly overexpressed in cell lines with known amplifications at 6p22 and defective RB1 (Table S1) like 5637 and HT-1376 (Figure 2a, light grey bars). The respective primer assay detected E2F3 isoforms A and B simultaneously (Table S2). Expression of ANRIL full length transcript and 5’-truncated variants significantly correlated with *E2F1* expression, but not with that of *E2F3* (Figure 2b).

To further elucidate the effect of both transcription factors on ANRIL expression, *E2F1* and *E2F3* expression was modulated either by siRNA-mediated knockdown or by ectopic expression in five different UC cell lines. Although ectopic expression and siRNA knockdown were highly efficient (Figure 2d,f), neither increased nor decreased expression of E2F1 or E2F3 resulted in prominent changes of ANRIL expression (Figure 2c,e). The only significant change was observed upon downregulation of *E2F1* in J82 cells (Figure 2c, *p* ≤ 0.05). This cell line displayed the highest expression levels of ANRIL and of *E2F1*. Complete loss of E2F1 protein expression (Figure 2d) resulted in reduced ANRIL expression (Figure 2c), concurring with the results from correlation analysis indicating a positive relation between both molecules.

### 2.3. ANRIL Is Not Involved in Epigenetic Silencing of the *INK4* Tumor Suppressor Locus in UC

**Figure 3:**
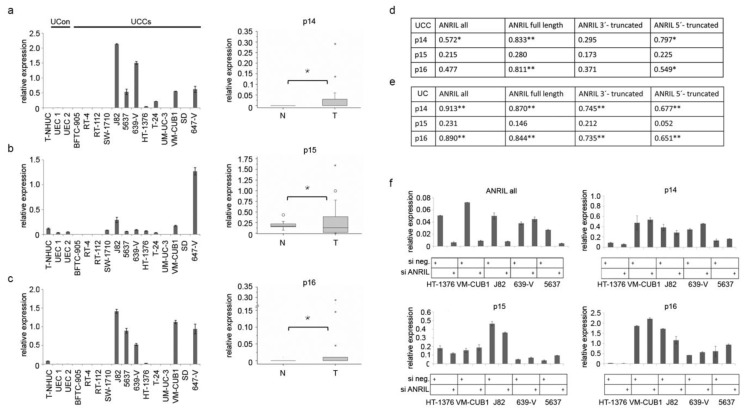
(**a**–**c**) Expression profiles of the three TS from the *INK4*/*ARF* locus were generated by qRT-PCR in UCCs (left panels) and tissue set 1 (boxplot graphs right panel; * *p* ≤ 0.05; T: *n* = 19; N: *n* = 10). Expression of different ANRIL variants was correlated with expression of the three TS in cell lines (**d**) and tissues (**e)** (** *p* ≤ 0.01; * *p* ≤ 0.05). (**f**) Knockdown of full length and truncated ANRIL variants by siRNA (top left panel) did not result in significant expression changes of the three TS in four different UCCs. SiRNA knockdown of ANRIL in 639-V cells was less efficient.

Next, we sought to explore the regulatory functions of ANRIL in UC, especially those of the truncated isoforms. To determine the impact of ANRIL on transcriptional activity of the three TS encoded by the *INK4*/*ARF* locus, we established the expression profiles of *p14, p15* and *p16* in UC cell lines and tissues compared to normal controls (Figure 3a–c).

While expression of all three TS factors was repressed in normal controls, UC cell lines without deletions in the *INK4*/*ARF* genes displayed detectable expression of *p14* and *p16*. *p15* expression was not significantly altered in most cell lines (Figure 3b). As expected, expression in UC tissues was also heterogeneous. While a number of samples lacked expression, others expressed the genes at variable levels. Highly increased levels were only detectable in individual samples, which notably also expressed ANRIL strongly. Calculation of Pearson correlation revealed significant associations between all types of ANRIL isoforms with *p14 or* *p16*, but not with *p15* in UC cell lines (Figure 3d) and tissues (Figure 3e). Unexpectedly, expression levels of TS and ANRIL transcripts were positively rather than inversely correlated.

To further study this relation we performed siRNA knockdown experiments. Knockdown of overall ANRIL expression was highly efficient in four of five cell lines, *i.e.*, HT-1376, VM-CUB1, J82, 5637, but not in 639-V (Figure 3f, top left panel). Not only expression of full length ANRIL, but also of truncated ANRIL isoforms was abolished by this siRNA knockdown (Figure S2e). However, no significant reduction in expression of any tumor suppressor transcript was observed (Figure 3f). Individual samples displayed slight expression changes following ANRIL knockdown, e.g., a reduction of *p15* and *p16* expression in high expressing J82 cells. Taken together, our data suggests that any regulatory function of ANRIL with regard to *INK4* TS may be rather stimulatory than inhibitory in UC.

As the *INK4* locus is implicated in regulation of self-renewal and differentiation, we also analyzed whether expression levels of cytokeratin differentiation markers (*CK14*, *CK5*, *CK20*) were altered by ANRIL knockdown. Like for *INK4*/*ARF* TS, no significant changes occurred (Figure S2a).

In order to validate our expression data for *INK4*/*ARF* TS, we collected the expression profile of the lncRNA PVT1 in UC cell lines, which was very recently reported to repress expression of genes encoded by the *INK4* locus in gastric cancer [33]. PVT1 was upregulated in some UC cell lines and tissues. As expected, we observed a negative association between expression of the lncRNA PVT1 and tumor suppressor genes, in particular for *p14* and *p16* in UC cell lines (Figure S2b).

Data obtained so far suggested that the lncRNA ANRIL does not have major repressive regulatory effects on the *INK4* locus in UC. If it exerts any regulatory effect *in cis* it is rather of a stimulatory nature. Thus, we sought to extend these results in functional assays by studying proliferation activity and senescence subsequent to knockdown of ANRIL.

**Figure 4:**
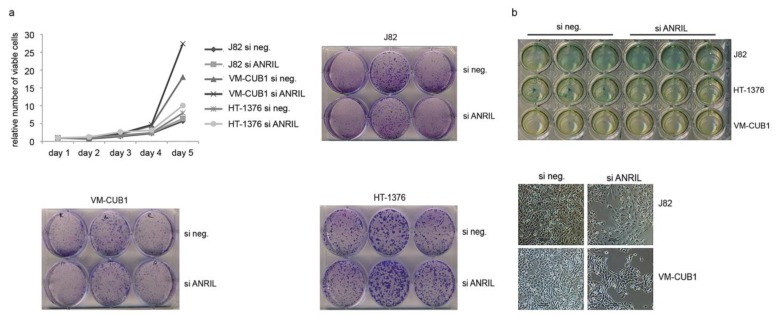
(**a**) Subsequent to siRNA-mediated knockdown of ANRIL (see Figure S2c for efficiency) proliferation activity was measured in three different cell lines. Representative results of multiple MTT assays are shown. Similarly, no difference in clonogenicity was observed. (**b**) Induction of senescence was analyzed by beta-galactosidase (SA-βgal) staining.

ANRIL knockdown (Figure S2c) elicited neither significant changes in proliferation (Figure 4a top left panel) and clonogenic capacity (Figure 4a), nor in the abundance of senescent cells (Figure 4b) in three UC cell lines.

### 2.4. ANRIL Isoforms Interact with PcG Protein CBX7 in UC Cells, But Not with SUZ12

Variations in regulatory effects of lncRNAs between tissues are expected to some extent as they are understood to function in fine-tuning of tissue- or context-dependent processes. This may result from tissue-dependent alterations, e.g., changes in subcellular localization of the lncRNA, abundance of interaction partners and binding capacities of the lncRNAs. The latter could particularly apply to truncated isoforms that may have lost binding sites, e.g., 3’-truncated variants of ANRIL, which appeared to be the predominantly expressed isoforms in UC (Figure 1 and Figure S1). Accordingly, such changes could be one mechanism leading to apparent loss of the negative regulatory function of ANRIL on the *INK4*/*ARF* locus in UC.

**Figure 5:**
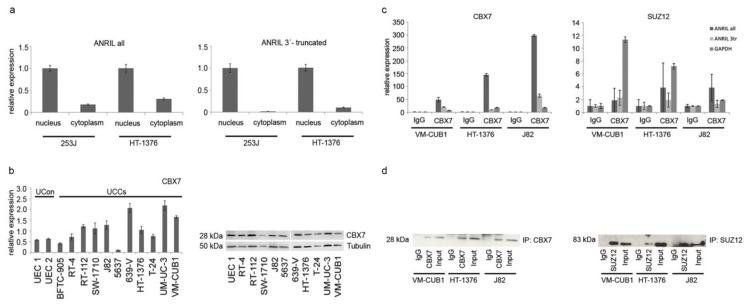
(**a**) ANRIL was mainly localized in the nucleus of UCCs as determined by qRT-PCR for all transcripts (left panel) and overexpressed 3’-truncated variants in particular (right panel) subsequent to fractionated extraction of RNA. Purity of nuclear fractions was checked by measuring the antisense transcript LIT1 as a control (Figure S2d). (**b**) Abundance of the interaction partner CBX7 in UCCs was checked on the RNA (left panel) and protein level (right panel). (**c**,**d**) Direct interaction between lncRNA ANRIL and PcG proteins was determined by RNA immunoprecipitation (RIP) analysis. Although both proteins were sufficiently precipitated (**d**), only interaction of ANRIL with CBX7 was detectable by qRT-PCR ((**c**), left panel), but not with SUZ12 above background levels (**c**, right panel). Results from IgG isotype controls were set as 1. GAPDH served as another background control. Both full length (included in “all” assay, dark grey bar) and 3’-truncated ANRIL isoforms (light grey bar) interacted with CBX7 protein.

We therefore first investigated whether ANRIL was still predominantly localized in the nucleus of UC cells (Figure 5a). The antisense transcript LIT1 originating from the *CDKN1C* tumor suppressor locus on Chr. 11p15 is well known to be exclusively localized in the nucleus [34], and served as a positive control for purity of fractionated RNA (Figure S2d). ANRIL isoforms, in particular the overexpressed 3’-truncated isoforms, were also mainly localized in the nucleus of UC cells (Figure 5a).

Established protein interaction partners of ANRIL are CBX7 in PC-3 cells and SUZ12, e.g. in WI38 cells. We already observed in our previous study on the function of lncRNA HOTAIR in UC [35] that SUZ12 is overexpressed in all investigated UC cell lines compared to normal urothelial cells. Likewise, CBX7 was significantly overexpressed in 7/11 UC cell lines (Figure 5b). Thus, with the exception of 5637 cells, four of the five cell lines that were used to study the regulatory effects of ANRIL by knockdown experiments displayed high abundance of both known interaction partners of ANRIL.

Finally, we studied the direct interaction between ANRIL and both PcG proteins in UC cells by RNA immunoprecipitation (RIP; Figure 5c,d). Initially, we validated our protocol for RIP analysis using the prostate cancer cell line PC-3. In these cells ANRIL was reported to interact with CBX7 [15]. Indeed, we found the full length ANRIL transcript, but also 3’-truncated variants bound to CBX7 (data not shown). Interestingly, when we analyzed UC cell lines, we discovered that significant amounts of ANRIL transcripts, including 3’-truncated isoforms were bound to CBX7 protein, but not to the SUZ12 protein (Figure 5c). Following SUZ12 precipitation ANRIL levels did not exceed background levels in three different UC cell lines in repeated experiments. Interaction with 5’-truncated variants was not analyzed, as such isoforms did not appear to be relevant in UC tissues.

## 3. Discussion

The lncRNA ANRIL is encoded within one of the most important tumor suppressor loci, which has been reported to be associated with various benign and malignant diseases including UC. A prominent function of ANRIL appears to be the repression of the tumor suppressor locus *INK4*/*ARF* in stem cells by suppressing replicative senescence and apoptosis [36]. Thus, in principle, aberrant ANRIL expression and activity constitute one mechanism to suppress this locus with consequences for cell cycle regulation, replicative senescence, apoptosis and self-renewal in various malignancies.

About 30% of UC carry mutations inactivating the *INK4*/*ARF* locus, most often homozygous deletions. In the present study, we investigated whether aberrant ANRIL expression might contribute to its inactivation in cases without genetic changes. In particular, we present for the first time expression data for ANRIL in UC for major groups of transcript isoforms with varying transcription starts and ends originating from 5’- and 3’-truncations. Some of them, in particular non-truncated and 3’-truncated variants have been shown to be functional in other systems [17,23]. However, at this time there is no comprehensive knowledge on which of the many ANRIL are functional in different contexts. In our study, we observed ANRIL to be overexpressed in many UC tissues. Interestingly, in our two tissue sample sets overexpression resulted mainly from an overexpression of 3’-truncated isoforms in about 50% of the patients. Most of the analyzed UC cell lines overexpressed ANRIL as well, except for those previously shown to harbor large homozygous deletions on chromosome 9p21 [4]. 

In tumor tissues ANRIL expression levels varied to some degree, likely due to genetic changes at the *INK4*/*ARF* locus. Variable levels of upregulated expression were also observed in studies on other cancer types including gastric, lung or hepatocellular cancer, where stratification into groups with low and high ANRIL expression revealed correlations with metastasis or survival [21,23,37]. In our study, ANRIL overexpression in UC was not associated with metastasis or survival. Concordantly, two very recently published studies on ANRIL in UC, did not report significant associations between ANRIL expression levels in UC tissues and clinicopathologic parameters. Martinez-Fernández *et al.* detected ANRIL expression prominently in non muscle-invasive UC, but this was neither significantly associated with tumor stage or grade, nor with tumor recurrence [38]. Zhu *et al.* reported overexpression of ANRIL in many UC compared to the corresponding normal tissue, but did not provide information on clinicopathologic characteristics of their UC sample set and their correlation with ANRIL expression levels [39]. Of note, previously published studies on ANRIL expression in cancer did not consider individual isoforms, which may account for conflicting results. However, diverse effects of aberrant ANRIL expression may be also explained by our results on the regulatory functions of ANRIL on the *INK4*/*ARF* tumor suppressor locus, which also differ from those reported in some other cancer types (see below).

While homozygous deletions on chromosome 9p21 straightforwardly explain the loss of ANRIL expression in some cancer tissues, the mechanisms leading to ANRIL overexpression in cancers with retention of the locus have not been clearly defined yet. The intergenic region between ANRIL and *p14^ARF^* harbors a bidirectional promoter, which was reported to contain E2F binding sites and to be responsive to E2F1 in HeLa, A549 and gastric cancer cells [23,27]. Accordingly, E2F1 dependent transactivation of ANRIL was observed as part of the DNA damage response in HCT116 cells [26]. Since E2F1 or E2F3 are commonly overexpressed in UC [29,31,32,40], we investigated the relation of both factors to ANRIL expression. In accordance with the literature, we found a positive correlation between expression of ANRIL and *E2F1*. However, neither siRNA-mediated knockdown nor ectopic overexpression of E2F1 led to prominent changes in ANRIL expression in different UC cell lines. The only exception were J82 cells, expressing high levels of ANRIL, in which downregulation of E2F1 diminished ANRIL expression. These data suggest that the stimulatory effect of E2F1 on ANRIL expression may be dose-dependent or require further tissue-dependent factors. No significant correlation was found between *E2F3* and ANRIL expression. This could be due to the lower number of UC tissues and cell lines overexpressing this factor [30,31]. Moreover, many of these UC lack functional pRB1 (Table S1), which has broad effects on the expression of the entire *INK4*/*ARF* locus and E2F factors [2,32]. However, neither knockdown nor overexpression of E2F3 affected ANRIL expression in UC cell lines. Taken together, these findings make E2F3 unlikely as a factor responsible for ANRIL overexpression in UC.

Several long noncoding RNAs have been shown to exert regulatory effects on neighboring protein-coding genes *in cis*, but some also regulate gene expression *in trans* as does ANRIL [13]. In cases in which lncRNAs contribute to gene repression, they often function by interacting with Polycomb Repressive Complexes PRC1 or PRC2 and recruiting them to the target locus. Specifically, ANRIL interacts with the CBX7 and SUZ12 proteins of the PRCs 1 and 2, respectively, and these interactions are thought to be involved in the repression of the *INK4*/*ARF* locus [41]. Accordingly, in the prostate cancer cell line PC-3 ANRIL was found to interact with CBX7 and the expression of *p16^INK4A^* was diminished [15]. However, ANRIL expression is not always inversely, but sometimes also positively correlated with that of all three TS. Binding of ANRIL to SUZ12 in WI38 cells regulated expression of *p15^INK4B^*, but not that of *p16^INK4A^* or *p14^ARF^* [16]. ANRIL knockdown in HCT116 cells increased *p15* and *p16*, whereas *p14* was only mildly affected [26]. Interestingly, ectopic overexpression of a newly identified alternatively spliced variant of ANRIL in HeLa cells did not change expression of any gene from the *INK*/*ARF* locus, but of many other genes involved in nuclear processes including chromatin regulation [27]. A further study also reported no regulatory effect of ANRIL expression on the *INK4* genes [17]. Moreover, these authors detected that a higher number of ANRIL target genes were rather stimulated than downregulated. In contrast, two studies observed a coordinated and positively correlation between the expression of ANRIL and *INK4* transcripts in a variety of normal and malignant tissues or cell lines [19,24]. In conclusion, regulatory effects of ANRIL on *INK4* and other target genes can be activating or repressing in a context-dependent manner and appear to be strongly tissue-specific. Such tissue-dependent variations may be a consequence of dose-dependency impinging on the abundance of and interactions between ANRIL binding partners [15,17]. Furthermore, the role of additional, possibly tissue-specific co-factors has to be considered [17]. Further, changes in subcellular localization of the long noncoding RNA might affect its interaction with protein partners. However, wherever investigated, ANRIL was mainly localized in the nucleus, e.g., in gastric or prostate cancer cells [15,23], like in UC cells in our study. Thus, delocalization of the lncRNA within UC cells to the cytoplasm is likely not a cause for its lack of effects on the *INK4*/*ARF* locus.

Concurring with the data published by Holdt *et al.* [17] and Burd *et al.* [24], we observed exclusively positive correlations between ANRIL isoforms and other transcripts from the *INK4*/*ARF* locus in UC cell lines, which were significant for *p14^ARF^* and *p16^INK4A^*. Expression of *p15* was not correlated, fitting the suggestion by Radvanyi *et al.* that *p15* and *p16* may be independently regulated in UC [42]. The same authors also reported *p15* and *p16* to be weakly expressed or lost in superficial, but enhanced in a subset of advanced UC [6]. These data are as well in accord with those results of our study showing rather coordinated expression of *INK4*/*ARF* transcripts in some UC tissues with ANRIL overexpression. The same authors postulated that a further unknown mechanism has to be invoked to explain loss of *p15* expression in cases without homozygous deletion and no detectable DNA hypermethylation. We conclude from our data that overexpression of neither the ANRIL nor PVT1 lncRNA is likely to constitute that mechanism in UC.

ANRIL enhances the aggressive phenotype of some cancer cells, e.g., proliferation activity of hepatocellular or gastric cancer cells, but mostly in cases where it also represses the *INK4*/*ARF* TS [15,23,43]. Accordingly, in keeping with the lack of inhibitory functions of ANRIL on this locus in UC, we did not observe significant changes in cell proliferation, clonogenicity or senescence in UC cells following ANRIL knockdown. In contrast, the study by Zhu *et al.* reported that ANRIL knockdown inhibited the growth of the UC cell line T24 and its subclone EJ mostly by impacting the intrinsic apoptotic pathway [39]. We have not seen evidence of increased apoptosis or decreased proliferation in three independent cell lines. To clarify this discrepancy, it will be important to investigate by which mechanism ANRIL regulates various components of the intrinsic apoptotic pathway such as BCL2 and BAX in T24 cells.

The positive correlations between ANRIL and *p14* may be explained by their shared bidirectional promoter [27]. Similarly, positive correlations between ANRIL and *INK4* genes do not necessarily indicate that ANRIL stimulates the transcription of the respective genes. Positive correlations could also result from a general open chromatin conformation allowing coordinated transcription of the lncRNA and the coding genes. Our results from ANRIL knockdown experiments and expression analysis of the three TS strengthen this hypothesis. Importantly, these findings clearly demonstrate that ANRIL expression *per se* is not sufficient to repress the locus, but depends on additional factors.

Known factors important in transcriptional repression by ANRIL are the interacting proteins CBX7 and SUZ12. Both proteins are significantly expressed in most cell lines from our panel and also reported to be commonly expressed in UC tissues [44], but expression of neither protein correlated significantly with expression of any ANRIL isoforms. Another explanation for varying functional effects of ANRIL could be that the various isoforms may differ in their ability to interact with repressor complexes. Data by Burd *et al.* [24] suggest that 9p deletions may change the prevalence of ANRIL isoforms towards variants containing rather distal exons 15–19, which they found to not correlate with *INK4* transcripts in human peripheral blood T-lymphocytes. A shift in the abundance of isoforms may therefore change the pattern of genes regulated by ANRIL and the extent and perhaps the direction of its influence. As we observed a shift towards 3’-truncated isoforms in UC, we wondered whether the isoforms differ in their capacity to bind known interaction partners like CBX7 and SUZ12. In RNA immunoprecipitation analyses both full length and 3’-truncated ANRIL transcripts strongly interacted with CBX7, but not with SUZ12. These data suggest that in UC, ANRIL interacts with PRC1 via CBX7, but not with the PRC2 (including SUZ12). At many loci PRC1 maintains gene repression following its initiation by histone methyltransferase components of PRC2 [45]. Accordingly, in cells where ANRIL repressed expression of *INK*/*ARF* genes, the respective promoter was also bound by SUZ12 [16]. Thus, transcriptional repression *in cis* might not be initiated by ANRIL in UC due to a lack of interaction between ANRIL and the initiation complex PRC2. As a consequence, positive correlations between *INK4*/*ARF* TS and ANRIL expression may result from either a transcription activating function of ANRIL or simply from the coordinated expression of transcripts from this locus.

## 4. Conclusions

The lncRNA ANRIL is overexpressed in many UC, but does not appear to exert inhibitory effects on *INK4*/*ARF* tumor suppressor genes. This lack of effect can be traced to tissue-dependent differences in binding activities between the lncRNA and PcG proteins, especially lack of interaction with SUZ12. Evidently, further hitherto unknown co-factors, likely to be tissue-specific, may determine whether ANRIL acts as repressor or activator of gene transcription. Evidently, a comprehensive identification of ANRIL interaction partners in different cell types is required to fully understand its function in normal and cancer tissues.

## 5. Material and Methods

### 5.1. Patients and Tissues

We initially used one set of tissue samples previously described elsewhere [46], comprising 10 benign bladder tissues and 19 UC tissues from patients aged 46 to 86 years (median age: 68 years). Tumor stages and grading according to the current UICC classification were as follows: 3 cases pTa G2, 2 cases pT2 G3, 2 cases pT3 G2, 9 cases pT3 G3 and 3 cases pT4 G3. Tissue samples were collected with patient informed consent and approval by the ethics committee of the medical faculty of the Heinrich Heine University, study number 3836 (issued 2012). The relation between ANRIL expression and clinicopathologic parameters was investigated in a second tissue sample set collected with patient informed consent and approval by the ethics committee of the medical faculty of the University Duisburg-Essen, Study Number 07-3537. This set consisted of 108 cancerous tissues and 7 normal tissues. For clinicopathologic parameters see Figure S1b.

### 5.2. Cell Culture and Transfection Experiments

Cell lines were provided by Dr. M.A. Knowles (Leeds, UK), Dr. J. Fogh (New York, USA), Dr. B. Grossman (Houston, USA) or by the DSMZ (Braunschweig, Germany). They were recently verified by DNA fingerprint analysis and regularly checked for mycoplasm contamination. Primary urothelial cells (UEC) were prepared from ureters after nephrectomy and were routinely maintained as described [47]. The TERT-immortalized normal human urothelial cell line TERT-NHUC was cultured in keratinocyte serum-free medium (Gibco, Darmstadt, Germany) supplemented with 0.25 ng/mL epidermal growth factor, 12.5 µg/mLbovine pituitary extract and 1:100 ITS (Gibco), 0.35 µg/mL(−)N-epinephrine and 0.33 mg/mL hydrocortisone (Sigma Aldrich, Taufkirchen, Germany).

For siRNA-mediated knockdown cells were transfected with 10 nM ANRIL siRNA (Lincode SMART-Pool #R-188105-00, Thermo Scientific Dharmacon, Darmstadt, Germany) compared to a non-targeting control pool (#D-001320-10, Thermo Scientific Dharmacon), siRNA against E2F1 (5’- GGACCUUCGUAGCAUUGCAtt; Ambion, Darmstadt, Germany), siRNA against E2F3 targeting both isoforms (5’- GCGAUCUCUUCGAUGCUUAtt, Ambion) or a non-targeting control (5’-AGGUAGUGUAAUCGCCUUG-5’; Ambion) using Lipofectamine RNAiMAX (Life Technologies, Darmstadt, Germany), according to the manufacturer’s recommendations.

For ectopic expression of *E2F1* or *E2F3* cells were transfected with pCMV-E2F1 (Addgene, Cambridge, MA, USA plasmid 24225) [48], pCMV-E2F3a (Addgene plasmid 37970) [49], pCMV-E2F3b (Addgene plasmid 37975) or empty vector using X-tremeGENE 9 DNA Transfection Reagent (Roche, Penzberg, Germany).

### 5.3. Assays for Cell Viability, Clonogenicity and Senescence

Cell proliferation was measured using the 3-(4,5-dimethylthiazol-2-yl)-2,5-diphenyltetrazolium bromide dye reduction assay (MTT, Sigma Aldrich). For clonogenicity assays cells were plated at low density into 6 cm dishes or 6 well plates 48 h after siRNA transfection. After 10 days, colonies were washed with PBS, fixed in methanol and stained with Giemsa (Merck, Darmstadt, Germany). Senescence-associated beta-galactosidase (SA-βgal) activity was detected by staining. SiRNA transfected cells were fixed for 5 min in 2% formaldehyde and 0.2% glutaraldehyde. Subsequent staining was performed at 37 °C for 4 h with fresh SA-β-Gal-staining solution (1 mg/mLX-Gal, 150 mM NaCl, 2 mM MgCl_2_, 5 mM K_3_Fe(CN)_6_, 5 mM K_4_Fe(CN)_6_). Images were taken using the NIS-Elements software with a Nikon Eclipse TE2000-S microscope (Nikon, Düsseldorf, Germany).

### 5.4. RNA Expression Analysis

Total mRNA was isolated using the RNeasy Mini Kit (Qiagen, Hilden, Germany) according to the manufacturer’s protocol. Fractionated RNA extraction was performed using the RNeasy Mini Kit (Qiagen) according to a specialized protocol [35]. One microgram of RNA as determined by spectrophotometery was reverse transcribed using the QuantiTect Reverse Transcription Kit (Qiagen). QRT-PCR was performed using QuantiTect SYBR Green RT-PCR Kit (Qiagen) with self-designed primers (Table S2) and the housekeeping gene *TBP* (TATA-box binding protein) as a reference. Reactions were carried out on the ABI PRISM^®^ 7500 HT (Life Technologies, Darmstadt, Germany) instrument. Primer assays were designed based on annotated data in the public databases www.ensembl.org and https://genome.ucsc.edu/. A Blast analysis was used to confirm that amplified sequences were unique.

### 5.5. RNA Immunoprecipitation

RNA immunoprecipitation (RIP) was performed as previously described [50]. Briefly, cells were lysed with standard RIPA lysis buffer supplemented with protease inhibitor (Sigma Aldrich) and RNAse inhibitor (Thermo Scientific, Darmstadt, Germany) according to the manufacturer’s instructions. Lysates were homogenized by means of a douncer. Magnetic protein G beads (Sure beads, Bio-Rad, München, Germany) were preincubated with the respective antibody for precipitation (CBX7: ab-21873; SUZ12: ab-175187, both Abcam, Cambridge, UK) for 1 h at room temperature. Control samples were incubated with rabbit IgG (Santa Cruz, Dallas, Texas, USA). Beads were washed with PBS-T before being rotated over night at 4 °C with the cell lysates. After washing with RIPA buffer beads were resuspended in Qiazol (Qiagen). Coprecipitated RNA was purified by chloroform extraction and precipitated with isopropanol.

### 5.6. Western Blot Analysis

Total protein was extracted by lysing the cells in a buffer containing 150 mM NaCl, 1% Triton X-100, 0.5% deoxycholate, 1% Nonidet P-40, 0.1% SDS, 1 mM EDTA, 50 mM Tris (pH 7.6), and protease inhibitor cocktail (10 μL/mL, Sigma-Aldrich) for 30 min on ice. Protein concentration was determined by BCA protein assay (Pierce) and samples were separated in SDS-page gels and transferred to PVDF membranes (Millipore, Darmstadt, Germany). Similarly, protein samples from RIP experiments were subjected to SDS-PAGE. The membrane was blocked with 5% non-fat milk in TBS-T (150 mM NaCl, 10 mM Tris, pH 7.4, and 0.1% Tween-20), washed and then probed with primary antibodies. Antibodies detected E2F1 (sc-251, Santa Cruz), E2F3 (sc-878, Santa Cruz) and α-Tubulin (ab-4074, Abcam) or CBX7 (ab-21873, Abcam) and SUZ12 (ab-175187, Abcam) for RIP experiments. After washing, the membrane was incubated with the suitable horseradish peroxidase-conjugated secondary antibody (Santa Cruz) for 1 h and exposed using ECL™ Quantum (Advansta, Menlo Park, CA, USA).

### 5.7. Statistical Analysis

Significance between groups was assessed by Student’s t-test. *p*-values of ≤0.05 were considered as significant (*), and *p* ≤ 0.01 as highly significant (**). Pearson correlation coefficients were calculated by means of the SPPS Statistics software version 21 (IBM, Armonk, NY, USA).

## Supplementary Files

Supplementary File 1
