# Supplementary material for: Truncated Isoforms of lncRNA ANRIL Are Overexpressed in Bladder Cancer, But Do Not Contribute to Repression of INK4 Tumor Suppressors
Source: Noncoding RNA. 2015 Dec 17;1(3):266–84. doi: 10.3390/ncrna1030266 (PMC5932551; doi:10.3390/ncrna1030266)
Supplement: Supplementary File 1 [file ncrna-01-00266-s001.pdf]

Supplementary Materials

Truncated Isoforms of lncRNA ANRIL Are Overexpressed in Bladder Cancer, But Do Not Contribute to Repression of INK4 Tumor Suppressors

Michèle J. Hoffmann, Judith Dehn, Johanna Droop, Günter Niegisch, Christian Niedworok, Tibor Szarvas and Wolfgang A. Schulz

a

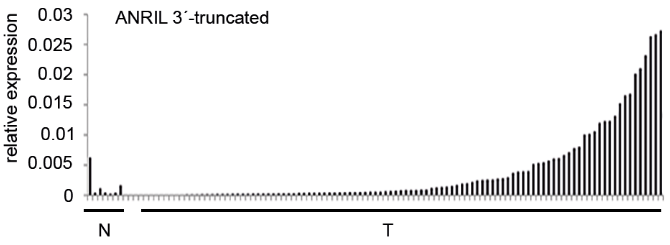

b

Variables	n	ANRIL expression median (range)	P
Age			
≤ 65	54	7.63 (0 - 273.01)	0.452
> 65	54	7.80 (0 - 5178.8)	
Gender			
Male	79	8.12 (0 - 266.54)	0.857
Female	29	6.05 (0 - 5178.7)	
Stage			
Ta	14	9.43 (0 - 5178.7)	
T1	13	7.14 (1.01 - 201.27)	
T2	17	8.57 (0 - 167.97)	
T3	44	6.35 (0.26 - 1233.7)	
T4	20	10.24 (0 - 273.01)	
Non-inv.	27	7.14 (0 - 5178.7)	0.804
Invasive	81	8.57 (0 - 1233.7)	
Grade			
G1	7	2.60 (0 - 231.82)	
G2	33	5.82 (0 - 5178.7)	
G3	68	9.12 (0 - 1233.7)	
Low-grade (G 1-2)	40	5.06 (0 - 5178.7)	0.125
High-grade (G 3)	68	9.12 (0 - 1233.7)	
Lyph node			
N0/Nx/M0/Mx	80	8.59 (0 - 273.01)	0.713
N + / M+	28	7.31 (0 - 1233.73)	
Smoking			
yes	61	7.14 (0 - 263.39)	0.907
no	29	5.17 (0 - 5178.7)	
unknown	18		
Control	7	4.30 (2.09 - 62.36)	0.726
Tumor	108	7.63 (0 - 5178.7)	

Figure S1. 3´-truncated ANRIL overexpression in UC tissue set 2(**a**) 3'-truncated ANRIL isoforms were overexpressed in a second set of UC tissues (T, *n* = 108) compared to normal controls (N, *n* = 7); (**b**) ANRIL expression in relation to clinicopathologic characteristics of UC Set 2.

Table S1. Summary of alterations in pRB1 in investigated UC cell lines according to mutation records from the CCLE database (www.broadinstitute.org/), Hurst *et al.* [32] and own data on protein expression.

UC cell line	Alteration in pRB1
RT-4	no reported changes
BFTC-905	no reported changes
RT-112	no reported changes
SW-1710	no reported changes
J82	splice site SNP
5637	nonsense mutation
639-V	nonsense mutation
HT-1376	nonsense mutation
T-24	no reported changes
UM-UC-3	no reported changes
VM-CUB1	no reported changes
SD	no reported changes
647-V	nonsense mutation

Table S2. Oligonucleotide sequences for named targets. Forward and reverse primer sequences are given in 5’ to 3’orientation.

Target	Sequence
ANRIL all	gcc tca ttc tga ttc aac agc
	agt act gac tcg gga aag gat tc
ANRIL 3’-truncated	gct tca ttc tat acc agg atc ca
	aag cag gta tca ttc tcc tca aa
ANRIL 5’- truncated	gaa ttt tga cag att ggc ttc
	tct gga ctg tga gac ata gat ttt
ANRIL full length	gaa ctc cca ggc tca aac c
	cct tcc ata cca tag tgc gtt ag
TBP	aca aca gcc tgc cac ctt a
	gaa tag gct gtg ggg tca gt
E2F1	cct cac cac aga tcc cag c
	atg ctc cag gag cga gtc g
E2F3	acg tct ctt ggt ctg ctc ac
	tct taa tga ggt gga tgc ct
$p14^{ARF}$	gtg gcc ctc gtg ctg atg
	cag cag ctc cgc cac tc
$p15^{INK4B}$	gcg ggg act agt gga gaa gg
	gcc tcc cga aac ggt tga
$p16^{INK4A}$	caa cgc acc gaa tag tta cg
	agc acc acc agc gtg tc
LIT1	ccc tgc tgt gcc ttc agc cc
	cca ggc tgc ctc acc caa cg
GAPDH	tcc cat cac cat ctt cca
	cat cac gcc aca gtt tcc
PVT1	ggc cag aag gag att aaa aa
	atg gtt cca cca gcg tta t

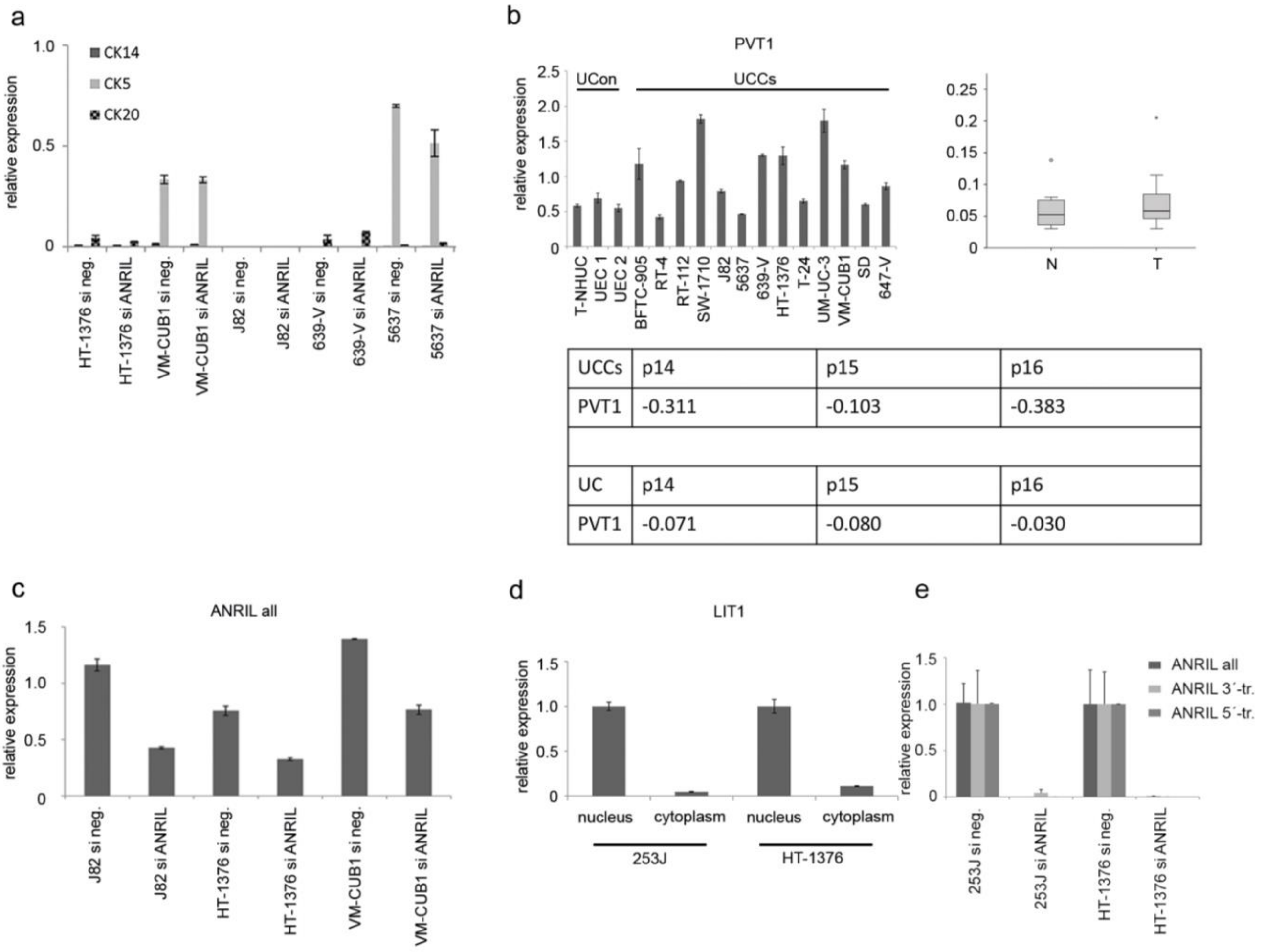

Figure S2. Further results from RNA exptression analysis. (**a**) Expression levels of the urothelial differentiation markers Cytokeratin (CK) 14, 5 and 20 were determined by qRT-PCR subsequent to siRNA knockdown of ANRIL in five UC cell lines. (**b**) Expression of the lncRNA PVT1 was inversely correlated with that of the three TS in UC cell lines (UCCs) and tissues (UC). Expression of PVT1 was determined by qRT-PCR relative to *TBP* in cell lines (left panel) and tissue sample set 1 (boxplot, right panel). (**c**) Efficiency of ANRIL knockdown in experiments on proliferation activity and senescence induction was checked. (**d**) Purity of nuclear RNA fractions was demonstrated by measuring LIT1 RNA, which is well known to be exclusively localized in the nucleus. (**e**) Despite full length ANRIL also expression of truncated ANRIL isoforms was abolished by siRNA knockdown.
